# *Porphyromonas gingivalis* Mfa1 Induces Chemokine and Cell Adhesion Molecules in Mouse Gingival Fibroblasts via Toll-Like Receptors

**DOI:** 10.3390/jcm9124004

**Published:** 2020-12-10

**Authors:** Yuhei Takayanagi, Takeshi Kikuchi, Yoshiaki Hasegawa, Yoshikazu Naiki, Hisashi Goto, Kousuke Okada, Iichiro Okabe, Yosuke Kamiya, Yuki Suzuki, Noritaka Sawada, Teppei Okabe, Yuki Suzuki, Shun Kondo, Tasuku Ohno, Jun-Ichiro Hayashi, Akio Mitani

**Affiliations:** 1 Department of Periodontology, School of Dentistry, Aichi Gakuin University, 2-11 Suemoridori, Chikusaku, Nagoya, Aichi 464-8651, Japan; ag173d14@dpc.agu.ac.jp (Y.T.); hisashi@dpc.agu.ac.jp (H.G.); okakou@dpc.agu.ac.jp (K.O.); iichiro@dpc.agu.ac.jp (I.O.); k-yosuke@dpc.agu.ac.jp (Y.K.); yukip@dpc.agu.ac.jp (Y.S); ag173d11@dpc.agu.ac.jp (N.S.); ag183d03@dpc.agu.ac.jp (T.O.); ag193d11@dpc.agu.ac.jp (Y.S.); ag193d07@dpc.agu.ac.jp (S.K.); tasuku@dpc.agu.ac.jp (T.O.); jun1row@dpc.agu.ac.jp (J.-I.H.); minita@dpc.agu.ac.jp (A.M.); 2 Department of Microbiology, School of Dentistry, Aichi Gakuin University, 1-100 Kusumoto-cho, Chikusaku, Nagoya, Aichi 464-8651, Japan; yhase@dpc.agu.ac.jp (Y.H.); naikiy@dpc.agu.ac.jp (Y.N.)

**Keywords:** *Porphyromonas gingivalis*, Mfa1, Toll-like receptors, gingival fibroblast

## Abstract

*Porphyromonas gingivalis* Mfa1 fimbriae are thought to act as adhesion factors and to direct periodontal tissue destruction but their immunomodulatory actions are poorly understood. Here, we investigated the effect of Mfa1 stimulation on the immune and metabolic mechanisms of gingival fibroblasts from periodontal connective tissue. We also determined the role of Toll-like receptor (TLR) 2 and TLR4 in Mfa1 recognition. Mfa1 increased the expression of genes encoding chemokine (C-X-C motif) ligand (CXCL) 1, CXCL3, intercellular adhesion molecule (ICAM) 1 and Selectin endothelium (E) in gingival fibroblasts, but did not have a significant effect on genes that regulate metabolism. Mfa1-stimulated up-regulation of genes was significantly suppressed in *Tlr4* siRNA-transfected cells compared with that in control siRNA-transfected cells, which indicates that recognition by TLR4 is essential for immunomodulation by Mfa1. Additionally, suppression of *Tlr2* expression partially attenuated the stimulatory effect of Mfa1. Overall, these results help explain the involvement of *P. gingivalis* Mfa1 fimbriae in the progression of periodontal disease.

## 1. Introduction

Periodontitis is a chronic inflammatory disease caused by multiple periodontal pathogenic bacterial species [1]. Socransky et al. indicated that three bacteria, *Porphyromonas gingivalis*, *Tannerella forsythia,* and *Treponema denticola*, are the main cause of the onset and progression of periodontitis and they suggested that these three species be called “red complexes” [2]. 

Among the three species, *P. gingivalis* is the major pathogen associated with periodontitis. Riviere et al. found that *P. gingivalis* was more prevalent at diseased sites than at healthy sites in diseased subjects [3]. The number of *P. gingivalis* in plaque samples was associated with the plaque score and clinical attachment level [4]. From the perspective of oral flora, *P. gingivalis* is considered to act as a keystone pathogen that creates a dysbiosis between the host and the dental biofilm. This altered oral commensal microbiota is responsible for initiating pathological bone loss [5]. *P. gingivalis* expresses a number of potential virulence factors, including lipopolysaccharides, gingipines, and fimbriae [6]. 

Fimbriae are filamentous proteinaceous appendages on the surface of *P. gingivalis* bacteria that play a pivotal role in colonization through association with other bacteria and host tissues [7,8]. *P. gingivalis* ATCC strain 33,277 has fimbriae consisting of either FimA (*fimA* gene product) or Mfa1 (*mfa1* gene product), with apparent molecular masses of approximately 38 and 75 kDa, respectively [9]. 

Mfa1, encoded by the *mfa1* gene, is a structural subunit of a protein complex whose length varies from 60 to 500 nm [10]. In addition to the primary Mfa1 protein, mature fimbriae also have affiliated Mfa2–5 proteins. Mfa2 plays an anchor role, while Mfa3 can bind with Mfa1/2/4/5 in vitro to connect with other fimbrial subunits [11]. Recent data indicate that the C-terminal domain of Mfa1, rather than Mfa3, affects the aggregation and maturation of downstream fimbrial proteins [12].

Several studies have demonstrated different roles for FimA and Mfa1 fimbriae. FimA fimbriae act as an adhesive that mediates periodontal tissue colonization and host cell invasion [8,13,14]. They also induce inflammatory processes in periodontal tissues through several mechanisms [15,16,17]. FimA fimbriae promote early biofilm formation in a single species of *P. gingivalis*, whereas Mfa1 plays an inhibitory role in the formation of a homotypic biofilm in *P. gingivalis* [18]. However, although there are few reports on the host immune response to Mfa1 fimbriae, an Mfa1-deficient strain causes almost no alveolar bone resorption in a mouse model of oral infection [19]. 

The present study aimed to examine the effect of *P. gingivalis* Mfa1 stimulation on the immune and metabolic mechanisms of mouse gingival fibroblasts (MGFs) and to examine the effects of Toll-like receptor (TLR) 2 and TLR4 knock down on *P. gingivalis* Mfa1-stimulated MGFs. Our results show that Mfa1 fimbriae had a large effect on immunomodulation exerted by gingival fibroblasts, but did not have a significant effect on metabolic regulation. Our results also indicate that recognition of Mfa1 by TLR4 on MGFs is essential for the expression of genes related to cell migration and cell adhesion. Overall, these results help to explain how *P. gingivalis* Mfa1 fimbriae are involved in the progression of periodontal disease.

## 2. Materials and Methods

### 2.1. Cell Culture

MGFs were isolated from healthy gingival tissue from the palate of BALB/c mice which were purchased from CLEA Japan, Inc. (Tokyo, Japan). The MGFs were cultured in Minimum Essential Medium α (Thermo Fisher Scientific, Wilmington, DE, USA) containing 10% fetal bovine serum (Hyclone Laboratories Inc, Logan, UT, USA), 100 U/mL penicillin, and 100 μg/mL streptomycin at 37 ℃ in a CO_2_ incubator. When the cells reached confluency, they were separated by treatment with 0.25% trypsin-EDTA (Thermo Fisher Scientific) and collected by centrifugation. This study was approved by the Institutional Animal Care and Use Committees of Aichi Gakuin University (AGUD438; approved date 31 March 2019) and all animal experiments were conducted following the national guidelines and the relevant national laws on the protection of animals. Ultrapure lipopolysaccharide from *P. gingivalis* was purchased for experiments (InvivoGen, San Diego, CA, USA).

### 2.2. Purification of Mfa1 Fimbriae

In this study, we used *P. gingivalis* mutant strains derived from ATCC 33,277. Mfa1 fimbriae were purified from JI-1, in which *fimA* was deleted, as described previously [20,21]. Mfa1 fimbriae were also purified from *mfa5* mutant FMFA5, in which *mfa5* was disrupted by *ermF-B*, and genetic complementation strain FMFA5C, as described previously [21,22]. Briefly, *P. gingivalis* cells were collected from 2 L of culture, suspended in 40 mL of 20 mM Tris/HCl buffer at pH 8.0, supplemented with protease inhibitors (1 mM phenylmethylsulfonyl fluoride and 1 mM Nα-p-tosyl-L-lysine chloromethyl ketone). The cells were disrupted by a French press. Unbroken cells were removed by centrifugation. The supernatant was subjected to precipitation at 50% ammonium sulfate saturation. The precipitate was dialyzed with 20 mM Tris/HCl buffer at pH 8.0, and then applied to DEAE Sepharose Fast Flow chromatography (GE Healthcare Bio-Sciences AB, Uppsala, Sweden) with 50 mL of bed volume. After washing thoroughly with the buffer, sample was fractionated by a linear gradient elution with 400 mL of NaCl (0 to 0.3 M) in the buffer. Purity and identity were verified by sodium dodecyl sulfate-polyacrylamide gel electrophoresis (Bio-Rad Laboratories, Hercules, CA, USA) and transmission electron microscopy. Details are described in the Supplementary Material.

### 2.3. RT2 Profiler PCR Array Analysis

MGF (1 × 10^6^ cells/dish) were seeded onto 60-mm dishes. When the cells reached confluency, they were incubated for 2 hours in the presence or absence of 1 μg/mL Mfa1, FMFA5, FMFA5C, FimA, or LPS of *P. gingivalis*. Total RNA was then collected. Complementary deoxyribonucleic acid (cDNA) was synthesized using the RT^2^ First Strand Kit (Qiagen, Germantown, MD, USA) according to the manufacturer’s instructions and then applied to the Mouse Antibacterial Response RT^2^Profile PCR Array (Qiagen) and the Mouse Extracellular Matrix & Adhesion Molecules RT^2^Profile PCR Array (Qiagen). Amplification was performed using the RT^2^ SYBR Green/ROX qPCR master mix (Qiagen) and the StepOnePlus^TM^ Real-time PCR system (Thermo Fisher Scientific) with associated software (version 2.3; Thermo Fisher Scientific). CT values were transferred to an Excel file to build a table of CT values, which was then uploaded onto the data analysis web portal at http://www.qiagen.com/geneglobe. 

### 2.4. Real-Time PCR

MGFs (1 × 10^6^ cells/dish) were seeded onto 60-mm dishes. When the cells reached confluency, they were incubated for 2 hours in the presence or absence of 1 μg/mL JI-1, FMFA5, FMFA5C, FimA or LPS of *P. gingivalis*. Total RNA was then extracted with a Nucleospin RNA kit (Macherey-Nagel Inc., Bethlehem, PA, USA) according to the manufacturer’s instructions, and purity and concentration were assessed by calculating the A230/A260 and A260/A280 ratios using a NanoDrop Lite (Thermo Fisher Scientific). To quantify mRNA, quantitative PCR was performed using the TaqMan gene expression assay (Thermo Fisher Scientific) for mouse *Cxcl1* (Mm04207460_m1), *Cxcl3* (Mm01701838), *Icam1* (Mm00516023_m1), *Sele* (Mm00441278_m1), *Tlr2* (Mm00442346_m1) and *Tlr4* (Mm00445273_m1) with the TaqMan Universal PCR Master Mix (Thermo Fisher Scientific). mRNA levels were normalized to the level of eukaryotic 18S rRNA (Hs99999901_s1). Quantitative PCR was performed using the StepOnePlus Real-Time System. PCR conditions were 10 minutes at 95 °C, followed by 40 cycles of 15 seconds at 95 °C and 1 minute at 60 °C. The relative amounts of target mRNAs were determined by subtracting the cycle threshold (CT) value for 18S rRNA from that for the gene (ΔCT). Then, the ΔCT value for the control group was subtracted from that for the experimental group (ΔΔCT). The results are expressed as the fold change (2-ΔΔCT) between the mRNA levels of control and experimental groups, where ΔΔCT was calculated as follows: ((CT for the target mRNA − CT for 18S rRNA) for the experimental group)–((CT for the target mRNA − CT for 18S rRNA) for the control group).

### 2.5. siRNA Transfection 

MGFs were transfected with siRNA targeting TLR2 and TLR4 (Silencer Select Pre-designed siRNAs, Ambion, Austin, TX, USA) or non-targeting control siRNA (Ambion) using Lipofectamine RNAiMAX (Thermo Fisher Scientific) according to the manufacturer’s protocol. Twenty-four hours after transfection, cells were stimulated with 1 μg/mL Mfa1, FMFA5, FMFA5C, FimA or LPS of *P. gingivalis* for 2 hours. The cells were then collected and TLR2 and TLR4 protein levels were determined by flow cytometry. Similarly, gene expression levels were determined by Real-Time PCR.

### 2.6. Flow Cytometry

MGFs (1 × 10^6^ cells in 100 μL) were incubated with anti-mouse CD282 (TLR2) phycoerythrin (PE) (BioLegend, San Diego, CA, USA, Cat: 148604), anti-mouse CD284 (TLR4) phycoerythrin (PE) (BioLegend, Cat: 117605), or isotype control antibody phycoerythrin (PE) (BioLegend, Cat: 400508) and analyzed by flow cytometry using a MACSQuant analyzer and MACSQuantify software version 2.4 (Miltenyi Biotec, Tokyo, Japan).

### 2.7. Statistical Analysis

Data were analyzed using PASW Statistics software (version 18.0; SPSS Japan, Tokyo, Japan). Differences among groups were examined by one-factor analysis of variance (ANOVA) and Bonferroni’s multiple comparison test. Comparisons of two independent groups were performed using Student’s t-test. Data are expressed as the mean ± standard deviation (SD). Significance was accepted at *p* < 0.05. 

## 3. Results

### 3.1. Analysis of Antibacterial Response-Associated Genes in Gingival Fibroblasts to Various Fimbriae

**Figure 1:**
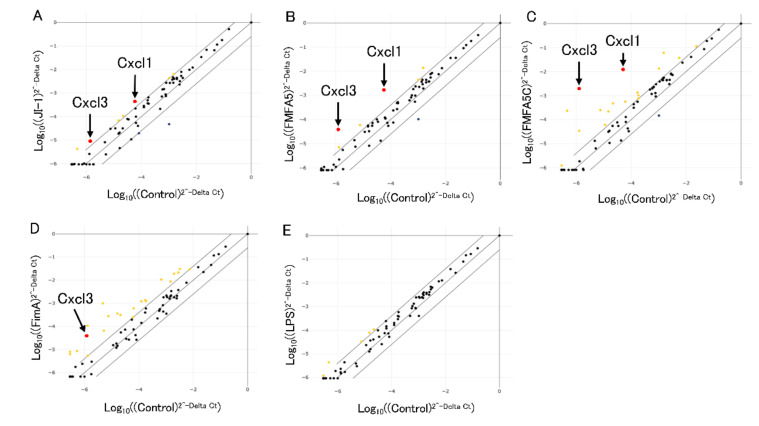
Analysis of antibacterial response-associated genes in mouse gingival fibroblasts in response to Mfa1, FimA, or *Porphyromonas gingivalis* LPS. Graphs show the fold changes of gene expression in cells stimulated with JI-1 (**A**), FMFA5 (**B**), FMFA5C (**C**), FimA (**D**), and LPS (**E**) compared with non-stimulated cells. *Cxcl1* and *Cxcl3* were up-regulated over 4-fold in JI-1, FMFA5 or FMFA5C-stimulated cells.

A mouse antibacterial response PCR array was used to investigate differences in the expression of 84 genes involved in bacteria-cell interactions. Figure 1 shows the fold changes in expression between control and 1 μg/mL various fimbriae or *P. gingivalis* LPS after stimulation for 2 hours. Among the 84 genes, *Cxcl1* and *Cxcl3* were upregulated in common by 4-fold in *JI-1*, *FMFA5* or *FMFA5C*-stimulated cells compared with non-stimulated cells (Figure 1A–C). Other genes that were elevated include *Nfkbia*, *Jun*, *Ccl5*, *Tlr6*, *Nod2* in *JI-1* stimulated cells, Nfkbia, Jun, Irf5, Birc3 in FMFA5-stimulated cells, and Hsp90aa1, Nfkb1, Nfkbia, Jun, Nod1, Slpi, Tirap, Rela, Ccl5, Tlr2, Irf5, Irf7, Nod2, Birc3, Lcn2 in FMFA5C-stimulated cells. Many genes (*Tnfrsf1a, Mapk1, Tollip, Irak1, Fadd, Map3k7, Map2k4, Slpi, Ripk2, Il18, Rela, Irak3, Card6, Camp, Irf5, Birc3, Slc11a1, Tlr9, Casp1, Crp*), including *Cxcl3* were up-regulated in FimA-stimulated cells compared with non-stimulated cells (Figure 1D). *P. gingivalis* LPS stimulation changed several genes expression (*Ccl5, Tlr6, Irf5, Nod2, Casp1*) compared with non-stimulated cells (Figure 1E).

### 3.2. Confirmation of PCR Array Data for Selected Antibacterial Response-Associated Genes by Quantitative Real Time-PCR

**Figure 2:**
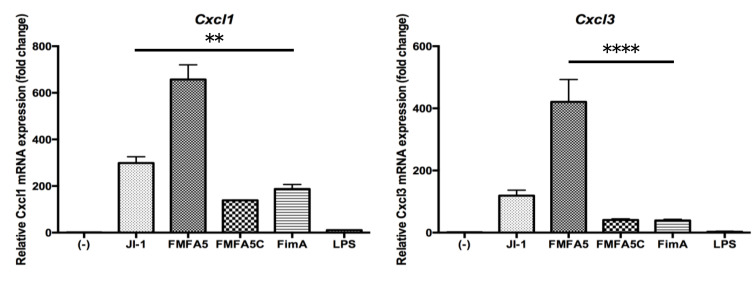
Mfa1 induces *Cxcl1* and *Cxcl3* in mouse gingival fibroblasts. Mouse gingival fibroblasts (MGFs) were cultured for 2 hours in the presence or absence of 1 μg/mL JI-1, FMFA5, FMFA5C, FimA, or LPS of *P. gingivalis* and then mRNA levels were examined using real-time PCR. Values are expressed as fold changes. Differences among groups were analyzed by one-way ANOVA. Data represent the mean + SD (*n* = 3). ** *p* < 0.01, **** *p* < 0.0001.

To validate the PCR array data, Real Time-PCR showed that JI-1 stimulation increased the gene expression of the cell migration factors, *Cxcl1* and *Cxcl3*, compared with FimA stimulation (Figure 2). Furthermore, the highest increase in gene expression was observed with FMFA5 stimulation (Figure 2).

### 3.3. Analysis of Extracellular Matrix and Adhesion Molecule-Associated Genes in Gingival Fibroblasts in Response to Various Fimbriae

**Figure 3:**
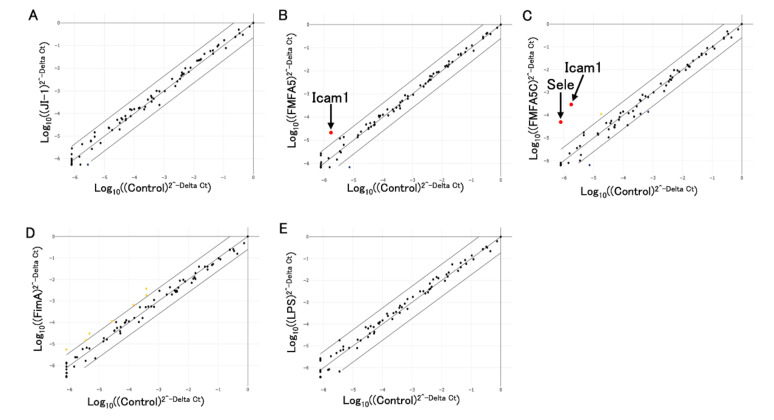
Analysis of extracellular matrix and adhesion molecule-associated genes in mouse gingival fibroblasts in response to Mfa1, FimA, or *P. gingivalis* LPS. Graphs show fold changes of gene expression in cells stimulated with JI-1 (**A**), FMFA5 (**B**), FMFA5C (**C**), FimA (**D**), and LPS (**E**) compared with non-stimulated cells. *Icam1* and *Sele* were up-regulated over 4-fold in FMFA5C-stimulated cells.

A mouse extracellular matrix and adhesion molecules PCR array was used to investigate differences in the expression of 84 genes involved in cell–cell and cell–matrix interactions. Figure 3 shows the fold changes in expression between control and 1 μg/mL various fimbriae or *P. gingivalis* LPS after stimulation for 2 hours. Among the 84 genes, *Icam1* expression was upregulated 4-fold in FMFA5-stimulated cells compared with non-stimulated cells (Figure 3B). FMFA5C stimulation similarly induced *Icam1* and also *Selectin E (Sele)* (Figure 3C). No obvious changes in gene expression were observed with JI-1, FimA or *P. gingivalis* LPS stimulation compared with unstimulated cells (Figure 3A,D,E).

### 3.4. Confirmation of PCR Array Data for Selected Extracellular Matrix and Adhesion Molecule-Associated Genes by Quantitative RT-PCR

**Figure 4:**
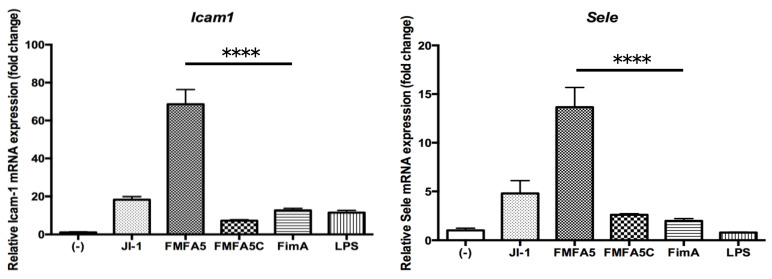
Mfa1 induces *Icam1* and *Sele* in mouse gingival fibroblasts. MGFs were cultured for 2 hours in the presence or absence of 1 μg/mL JI-1, FMFA5, FMFA5C, FimA, or LPS of *P. gingivalis* and then mRNA levels were examined using real-time PCR. Values are expressed as fold changes. Differences among groups were analyzed by one-way ANOVA. Data represent the mean + SD (*n* = 3). **** *p* < 0.0001.

Expression of cell adhesion factors, *Icam1* and *Sele*, was increased to a greater extent by JI-1 stimulation compared with FimA stimulation (Figure 4). The highest increase in gene expression was observed with FMFA5 stimulation (Figure 4).

### 3.5. Induction of Tlr2 and Tlr4 Gene Expression by Various Fimbriae and LPS Stimulation in Gingival Fibroblasts

**Figure 5:**
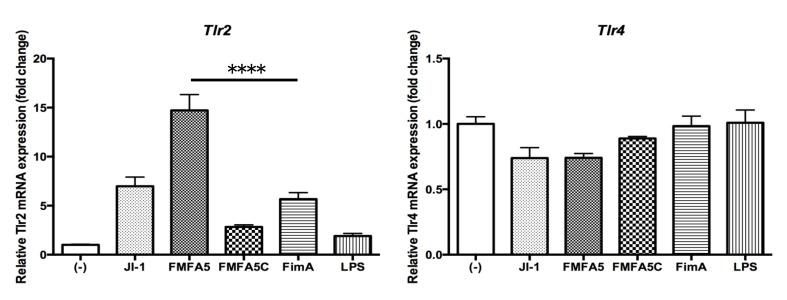
Mfa1 induces TLR2 but not TLR4 in mouse gingival fibroblasts. MGFs were cultured for 2 hours in the presence or absence of 1 μg/mL JI-1, FMFA5, FMFA5C, FimA or LPS of *P. gingivalis* and then mRNA levels were examined using real-time PCR. Values are expressed as fold changes. Differences among groups were analyzed by one-way ANOVA. Data represent the mean + SD (*n* = 3). **** *p* < 0.0001.

*Tlr2* showed the same change in expression as the other factors, such as *Cxcl1*, but *Tlr4*, the receptor for LPS, did not show any significant variation in gene expression in response to various stimulants (Figure 5).

### 3.6. Transfection of Tlr2 and Tlr4 siRNA into Gingival Fibroblasts

**Figure 6:**
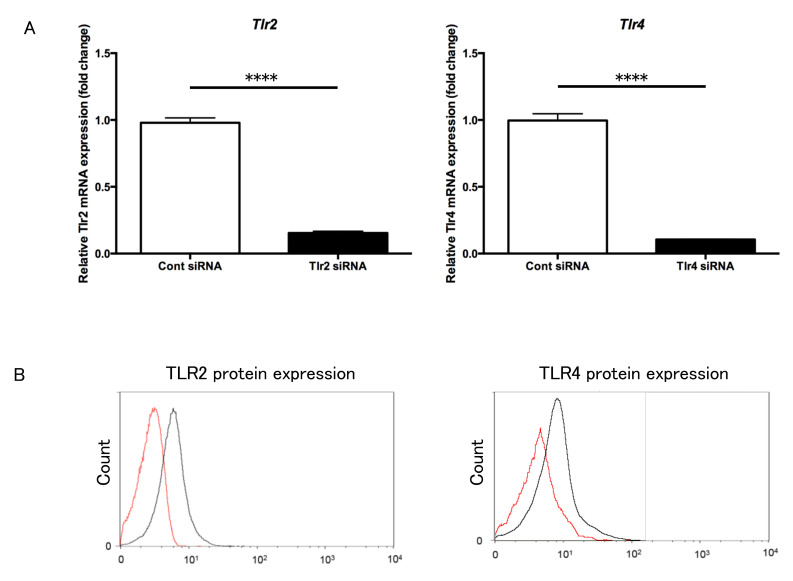
Suppression of *Tlr*2 and *Tlr4* in mouse gingival fibroblasts using siRNA. (**A**) *Tlr2* (left) and *Tlr4* (right) mRNA levels were examined using real-time PCR. *Tlr2* and *Tlr4* siRNA-transfected cells showed clear knockdown of *Tlr2* and *Tlr4* mRNAs, respectively, compared with control siRNA-transfected cells. (**B**) TLR2 (left) and TLR4 (right) protein levels were examined using flow cytometry. *Tlr2* or *Tlr4* siRNA transfected cells (red line) showed decreased levels of the respective receptors compared with control siRNA-transfected cells (black line). **** *p* < 0.0001.

*Tlr2* and *Tlr4*siRNA-transfected gingival fibroblast cells showed obvious knockdown of *Tlr2* and *Tlr4*mRNA, respectively, compared with control siRNA-transfected gingival fibroblast cells (Figure 6A). FACS analysis confirmed a decrease in the surface expression of *Tlr2* and *Tlr4* in the respective siRNA-transfected gingival fibroblast cells compared with control cells (Figure 6B).

### 3.7. Expression of Selected Genes in Tlr2 and Tlr4 siRNA-Transfected Cells

**Figure 7:**
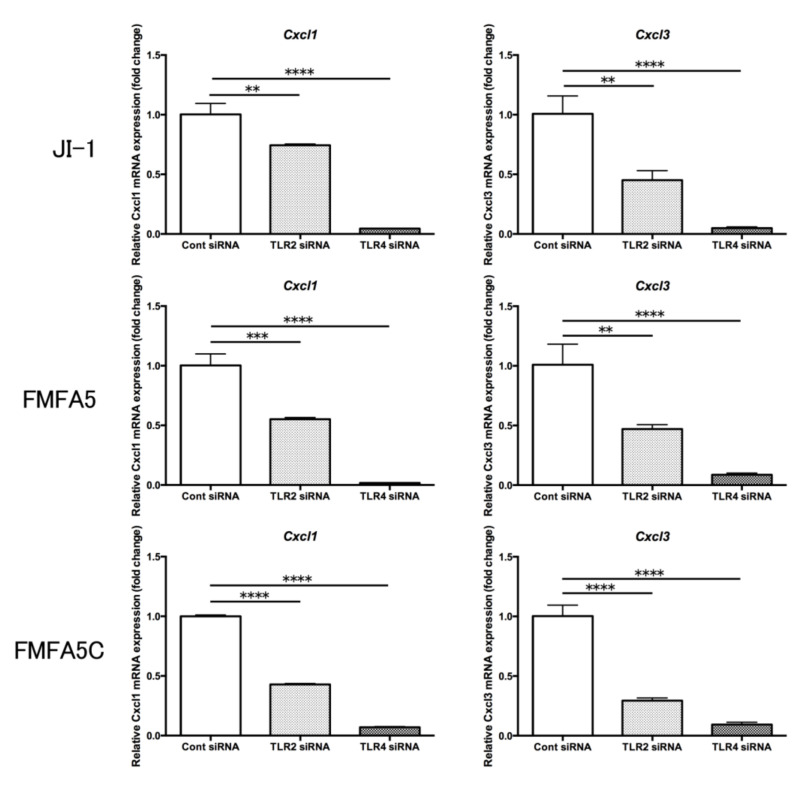

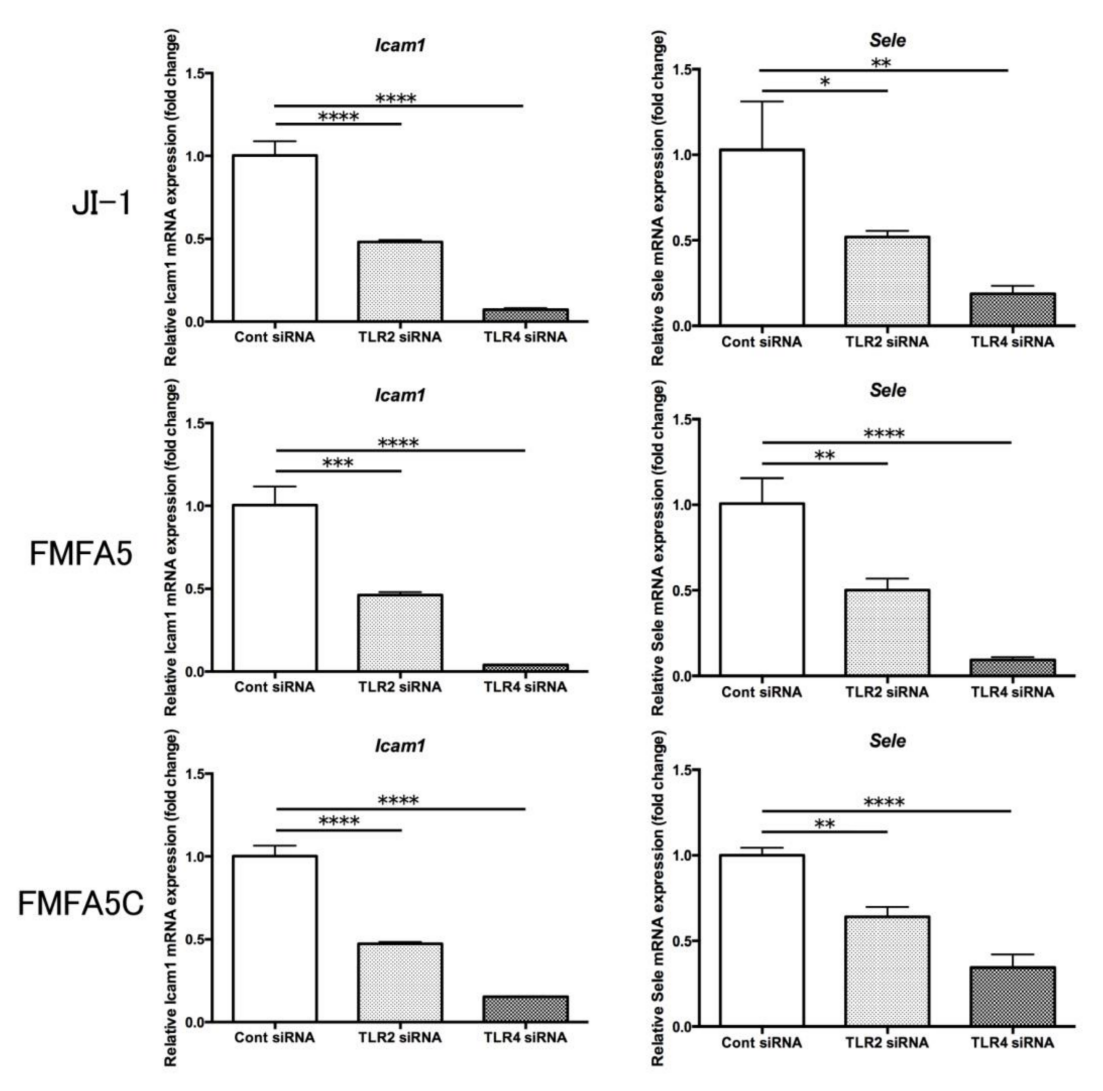
Involvement of TLR2 and TLR4 in the expression of Mfa1-induced cell migration and cell adhesion-related factors. *Tlr2* siRNA, *Tlr4* siRNA, or control siRNA-transfected mouse gingival fibroblasts were cultured for 2 hours in the presence or absence of 1 μg/mL JI-1, FMFA5, or FMFA5C and then mRNA levels were examined using real-time PCR. Values are expressed as fold changes. Differences among groups were analyzed by one-way ANOVA. Data represent the mean + SD (*n* = 3). * *p* < 0.05, ** *p* < 0.01, *** *p* < 0.001, **** *p* < 0.0001.

Expression of the cell migration-related factor and cell adhesion factor genes in JI-1, FMFA5, and FMFA5C-stimulated *Tlr4* siRNA-transfected cells were significantly suppressed compared with control siRNA-transfected cells (Figure 7). Also, the suppression of *Tlr2* expression partially attenuated the stimulation effect (Figure 7).

## 4. Discussion

In this study, we demonstrated that Mfa1 fimbriae stimulation markedly increased the expression of cell migration/cell adhesion-related genes in mouse gingival fibroblasts, and that the increase was more pronounced than with FimA stimulation. In addition, the ability of Mfa1 to regulate cell migration and cell adhesion was significantly attenuated in mouse gingival fibroblasts in which *Tlr4* expression was suppressed.

When the effect of JI-1 on gingival fibroblasts, which constitute the gingival connective tissue, was examined, a remarkable increase in the expression of *Cxcl1* and *Cxcl3*, which are involved in cell migration, was observed. Higher levels of CXCL1 were found in human and rat gingiva from periodontitis sites compared with periodontally healthy sites [23]. There was also a significant difference in the level of CXCL1 in gingival crevicular fluid between healthy and periodontitis subjects [24]. CXCL1 stimulates gingiva fibroblast migration [25] and it may be related to periodontal tissue healing. There is no literature reporting the relationship between CXCL3 and periodontitis, but its activity is similar to that of CXCL2, which may play an important role in the initiation of inflammation and subsequent periodontal tissue destruction [26], which suggests that it is involved in the progression of periodontitis. JI-1 stimulation produced a higher increase in *Cxcl1* and *Cxcl3* expression compared with FimA stimulation. FimA induces *Cxcl1* expression in mouse peritoneal macrophages [27]. It is possible that the immunomodulatory capacity of Mfa1 outperforms that of FimA for some factors in certain cell types. 

Next, we investigated which part of the fimbriae structure is important for the immunomodulatory ability of Mfa1. FMFA5, with the Mfa3–5 tip structure of JI-1 removed, and FMFA5C, with the tip structure was complemented in FMFA5, were compared for their ability to induce immunomodulation. Compared with JI-1, a markedly higher increase in gene expression was observed with FMFA5 stimulation, while that of FMFA5C was significantly reduced. From this result, it is indicated that the incomplete fimbriae deficient in tip structure increase the stimulation ability.

When we confirmed the effects of Mfa1 on extracellular matrix and adhesion molecules, we found a marked increase in the expression of the cell adhesion factor genes, *Icam1* and *Sele*. ICAM1 deficiencies increase susceptibility to and severity of alveolar bone loss after *P. gingivalis* infection [28]. Selectin Platelet/Selectin E-deficient mice exhibit spontaneous early onset alveolar bone loss [29]. FimA and Mfa1 induce ICAM1 and Selectin E in human aortic endothelial cells [30]. These fimbriae appear to have similar effects on gingival fibroblasts. Similar to the results for cell migration factors, a markedly higher increase in cell adhesion factor gene expression was observed with FMFA5 stimulation and the stimulation ability of FMFA5C was significantly reduced compared with JI-1 stimulation. These results indicate that the regulation of cell adhesion factors by Mfa1 is also greatly influenced by the Mfa1 molecule in the shaft portion.

Cells use pattern recognition receptors, such as TLRs, to recognize pathogen-associated molecular patterns (PAMPs). Ten TLRs have been identified in humans and 12 in mice and the host innate immune response to pathogens is largely mediated via TLR signaling [31]. TLR2 and TLR4 are the most widely studied extracellular innate receptors that recognize various PAMPs and are likely to play a role in the pathogenesis of periodontitis [32]. TLR2 has been shown to be important for *P. gingivalis* to produce inflammatory cytokines [33,34]. TLR4 recognizes LPS (from *Escherichia coli*), which is a bacterial cell wall component [31]. Uniquely, *P. gingivalis* LPS is recognized by both TLR2 and TLR4 [35]. The LPS_1435/1449_ and LPS_1690_ isoforms can produce opposite effects on TLR2 and TLR4 activation [36,37]. *P. gingivalis* LPS and *E. coli* LPS regulate cytokine production differently in human gingival fibroblasts [38]. The heterogeneity of *P. gingivalis* LPS might contribute to one of the strategies used by *P. gingivalis* to evade the innate host defense in gingival tissues [39]. FimA activates human peripheral blood monocytes via TLR2 and CD14 [40]. TLR2-dependent signaling leads to CD11b-CD18 activation in human monocytes upon recognition of FimA through CD14 [41]. However, the receptor for Mfa1 is still unclear. The gingival fibroblasts constitutively express TLR2 and TLR4 [42]. When we examined the expression of *Tlr2* and *Tlr4* genes in MGFs stimulated by various fimbriae and LPS, *Tlr2* showed the same changes in expression as the other factors, but *Tlr4* did not show any significant changes in expression in response to various fimbriae or LPS. Therefore, to clarify the receptor of Mfa1, MGFs in which *Tlr2* and *Tlr4* were knocked down were stimulated with Mfa1 and their reactivity was assessed. Control siRNA, *Tlr2* siRNA and *Tlr4* siRNA were introduced into MGFs, and cells were stimulated with JI-1, FMFA5, and FMFA5C. Gene expression of cell migration-related genes, *Cxcl1* and *Cxcl3*, and cell adhesion genes, *Icam1* and *Sele*, were analyzed. The suppression of TLR4 was significantly reduced by Mfa1 fimbriae stimulation. Also, suppression of *Tlr2* partially attenuated this stimulation. Recognition of Mfa1 by TLR4 is suggested to be essential for the expression of genes related to cell migration and cell adhesion. This result is different from previous reports in which anti-TLR2 antibody pretreatment significantly inhibited pro-inflammatory cytokines in mouse macrophages stimulated with Mfa1 fimbriae [43]. Conversely, Hajishengallis et al. reported that native Mfa1 induced proinflammatory cytokines in a CD14- and TLR2-dependent mode, which was likely due to a fimbriae-associated 12-kDa lipoprotein [44]. There is a similar report on FimA-like lipoproteins or lipopeptides associated with FimA that could, at least in part, account for signaling via TLR2 and subsequent TNF-α production in macrophages [45]. Furthermore, the following reports demonstrate the stimulation ability of LPS. A lipoprotein from *P. gingivalis* LPS was shown to be a principal component for TLR2-mediated cell activation [46]. A lipopolysaccharide preparation extracted from a *P. gingivalis* lipoprotein-deficient mutant showed a marked decrease in TLR2-mediated signaling [47]. Recombinant FimA stimulated cytokine release in THP-1 mononuclear cells via CD14 and TLR4 but not TLR2 [44], while recombinant FimA induced an inflammatory response via the TLR4/NF-kB signaling pathway in human peripheral blood mononuclear cells [48]. *P. gingivalis* lipid A and its synthetic counterpart activate cells through a TLR4-dependent pathway [46,49]. From our results, we speculate that wild-type purified Mfa1 is mainly recognized by TLR4, but TLR2 might recognize the lipoprotein of fimbriae and contributes to the overall action. In the future, it is necessary to confirm the reactivity of purified Mfa1 from which lipoprotein has been removed. 

There are several reports on the importance of TLR4 in periodontitis. TLR4- but not TLR2-mediated stimulation, was positively associated with plaque score and bleeding on probing score of teeth from which the plaque samples were taken [4]. The ratio of TLR4/TLR2-mediated stimulation activity was also positively associated with probing depth and clinical attachment level [4]. TLR4- but not TLR2-stimulation of subgingival plaque is associated with plaque index [50]. Therefore, TLR4 may play an important role in the progression of periodontitis, in which stimulation by Mfa1 may play a role. 

Recent reports suggest that intracellular DC-SIGN, an intracellularly expressed pattern recognition receptor, could be critical for recognition of Mfa1 by dendritic cells [51]. We assayed expression of DC-SIGN in MGFs after stimulation with various fimbriae, but we could not detect its expression. This may be because of differences between immunocompetent cells and periodontal tissue constituent cells. 

In conclusion, Mfa1 fimbriae have a significant effect on immunomodulation in gingival fibroblasts of periodontal tissue. We also suggest that recognition of Mfa1 by TLR4 on MGFs is essential for the expression of genes related to cell migration and cell adhesion. More detailed analysis, such as using an animal infection model, is needed to assess the immunomodulatory capacity of Mfa1 fimbriae in the progression of periodontitis.

## Supplementary Materials

The following are available online at https://www.mdpi.com/2077-0383/9/12/4004/s1, Figure S1: Purity of Mfa1 fimbriae.
