# Supplementary material for: Porphyromonas gingivalis Mfa1 Induces Chemokine and Cell Adhesion Molecules in Mouse Gingival Fibroblasts via Toll-Like Receptors
Source: J Clin Med. 2020 Dec 10;9(12):4004. doi: 10.3390/jcm9124004 (PMC7764148; doi:10.3390/jcm9124004)
Supplement: Supplementary file 1 [file jcm-09-04004-s001.pdf]

Supplementary Material

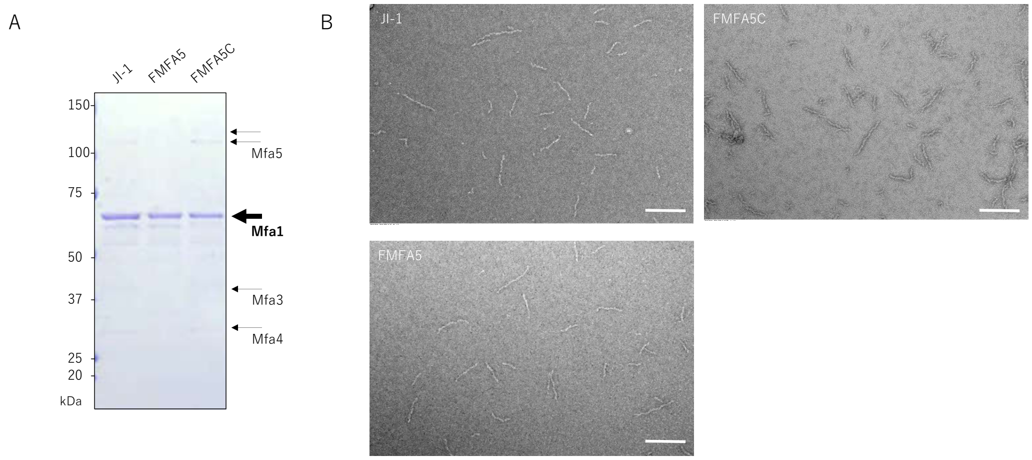

Figure 1. Purity of Mfa1 fimbriae. (A) Sodium dodecyl sulfate-polyacrylamide gel electrophoresis (SDS-PAGE) and Coomassie brilliant blue (CBB) staining of the purified Mfa1 fimbriae (1 µg/lane). The Mfa1 band indicated with a bold arrow was clearly detected. Mfa3, Mfa4, and Mfa5 are indicated with thin arrows as the minor components of Mfa1 fimbriae. (B) Electron micrograph of the purified Mfa1 fimbriae. Mfa1 fimbriae were negatively stained with 2% uranyl acetate. Scale bar, 100 nm.

Supplementary Methods

Sodium dodecyl sulfate-polyacrylamide gel electrophoresis (SDS-PAGE) and protein staining

The samples of the purified fimbriae were mixed with a loading buffer consisting of 50 mM Tris/HCl, pH 6.8, 1% (w/v) SDS, 0.5 M 2-mercaptoethanol, 10% (w/v) glycerol, and 0.01% (w/v) bromophenol blue (all at final concentrations), and denatured by heating at 100℃ for 5 min. Then, the samples were loaded onto an SDS-PAGE gel consisting of 11% or 5–20% gradient polyacrylamide (SuperSep™ Ace, Wako, Osaka, Japan). After electrophoresis, protein bands were visualized by staining with Coomassie Brilliant Blue R-250(Tokyo Chemical Industry, Tokyo, Japan).

Transmission electron microscopy

The sample of the purified Mfa1 fimbriae were placed on a carbon-film grid, negatively stained with 2% uranyl acetate and observed by transmission electron microscopy (HITACHI H-7600, Hitachi, Tokyo, Japan).

Supplementary Legend

Figure 1. Purity of Mfa1 fimbriae. (A) Sodium dodecyl sulfate-polyacrylamide gel electrophoresis (SDS-PAGE) and Coomassie brilliant blue (CBB) staining of the purified Mfa1 fimbriae (1μg/lane). The Mfa1 band indicated with a bold arrow was clearly detected. Mfa3, Mfa4, and Mfa5 are indicated with thin arrows as the minor components of Mfa1 fimbriae. (B) Electron micrograph of the purified Mfa1 fimbriae. Mfa1 fimbriae were negatively stained with 2% uranyl acetate. Scale bar, 100 nm.

Acknowledgements

We thank Hanaichi Ultrastructure Research Institute, Okazaki, Japan for performing transmission electron microscopy.
